# The genome sequence of a carabid beetle, *Nebria salina* (Fairmaire & Laboulbène, 1854)

**DOI:** 10.12688/wellcomeopenres.19372.1

**Published:** 2023-06-14

**Authors:** Olga Sivell, Duncan Sivell, Natural History Museum Genome Acquisition Lab, Darwin Tree of Life Barcoding collective, Wellcome Sanger Institute Tree of Life programme, Wellcome Sanger Institute Scientific Operations: DNA Pipelines collective, Tree of Life Core Informatics collective, Darwin Tree of Life Consortium

**Affiliations:** 1 Natural History Museum, London, England, UK

**Keywords:** Nebria salina, a carabid beetle, genome sequence, chromosomal, Coleoptera

## Abstract

We present a genome assembly from an individual female *Nebria salina* (a carabid beetle; Arthropoda; Insecta; Coleoptera; Carabidae). The genome sequence is 256.7 megabases in span. Most of the assembly is scaffolded into 21 chromosomal pseudomolecules, including the assembled X sex chromosome. The mitochondrial genome has also been assembled and is 24.7 kilobases in length. Gene annotation of this assembly on Ensembl identified 10,671 protein coding genes.

## Species taxonomy

Eukaryota; Metazoa; Ecdysozoa; Arthropoda; Hexapoda; Insecta; Pterygota; Neoptera; Endopterygota; Coleoptera; Adephaga; Caraboidea; Carabidae; Nebriinae; Nebriini; *Nebria; Boreonebria*; *Nebria salina* (Fairmaire & Laboulbène,, 1854) (NCBI:txid878211).

## Background

*Nebria salina* (Coleoptera, Carabidae) is widespread in Britain, although less common and more locally distributed than the closely related *Nebria brevicollis* (Fabricius, 1792) ( Lindroth, 1974; Luff, 1998; Luff, 2007). These two *Nebria* species are very similar in appearance, with lengths ranging from 11 to14 mm, and black in colour except for the reddish-brown antennae, tibiae and tarsi. *Nebria salina* lacks hairs on the dorsal surface of the hind tarsi ( Luff, 1981; Luff, 2007), which is the main character separating it from *N. brevicollis*. The beading on the edge of the pronotum is comparatively narrower in *N. salina* ( Duff, 2012; Hůrka, 1996; Walters, 2010), and, at higher magnification, the elytral microsculpture is coarser and less transverse than in *N. brevicollis* ( Lindroth, 1974; Luff, 1981; Luff, 2007).

*Nebria salina* prefers drier, less productive soils such as sand dunes and heaths, or upland grasslands and moraines ( Anderson *et al.*, 2000; Duff, 2012; Luff, 1998). It may occur together with the more eurytopic *N. brevicollis* in some marginal habitats such as woodland edge ( Lindroth, 1985). James (2018) advises that records of *N. salina* from nutrient-rich soils that lack voucher specimens should be treated with caution.

*Nebria salina* adults are nocturnal and prey on a variety of invertebrates of suitable size such as beetles, flies, spiders, mites and springtails ( Telfer & Butterfield, 2004). The larvae are also active surface predators ( Lindroth, 1974). *Nebria salina* has an annual life cycle, spending the winter as a larva and emerging as an adult in the spring. After a period of spring activity, the adults enter a summer diapause but become active again in the autumn when they mate, producing the generation of larvae that will live through the next winter ( Lindroth, 1985; Telfer & Butterfield, 2004).

The high-quality genome sequence described here is the first one reported for *Nebria salina*. It has been generated as part of the Darwin Tree of Life project. It will aid research into the taxonomy, biology and ecology of the species.

## Genome sequence report

**Figure 1.**
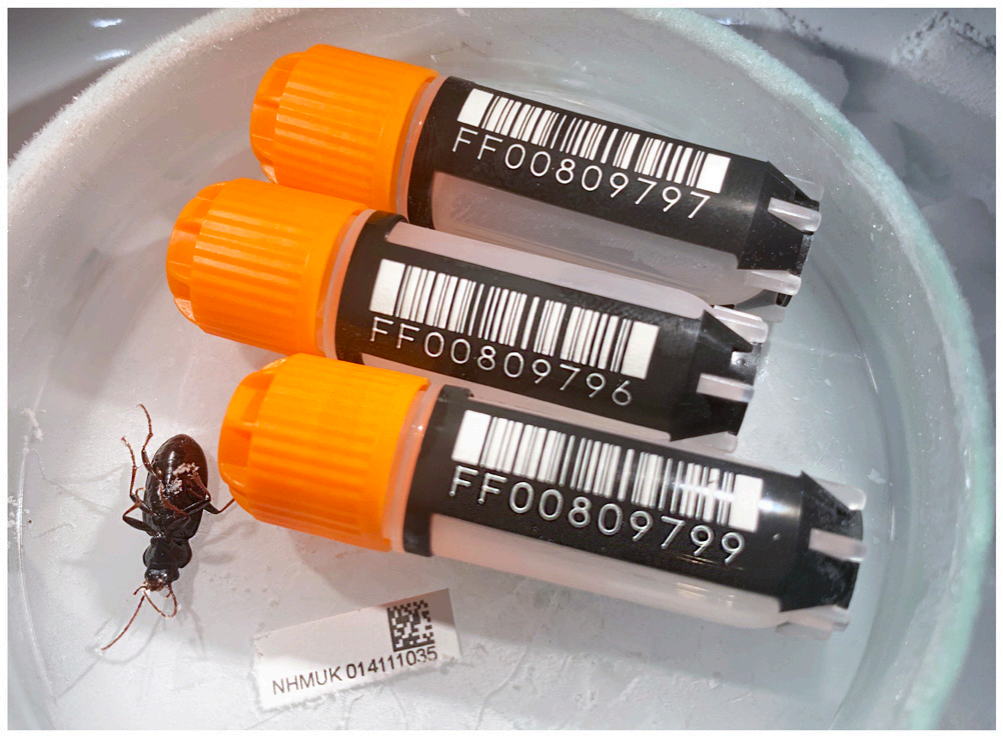
Photograph of the *Nebria salina* (icNebSali1) specimen used for genome sequencing.

The genome was sequenced from one female *Nebria salina* ( Figure 1) collected from Wigmore Park, Luton, England (51.88, –0.37). A total of 45-fold coverage in Pacific Biosciences single-molecule HiFi long reads and 51-fold coverage in 10X Genomics read clouds were generated. Primary assembly contigs were scaffolded with chromosome conformation Hi-C data. Manual assembly curation corrected 172 missing joins or mis-joins, and removed two haplotypic duplications, reducing the assembly length by 0.18% and the scaffold number by 19.56%, and increasing the scaffold N50 by 147.22%.

**Figure 2.**
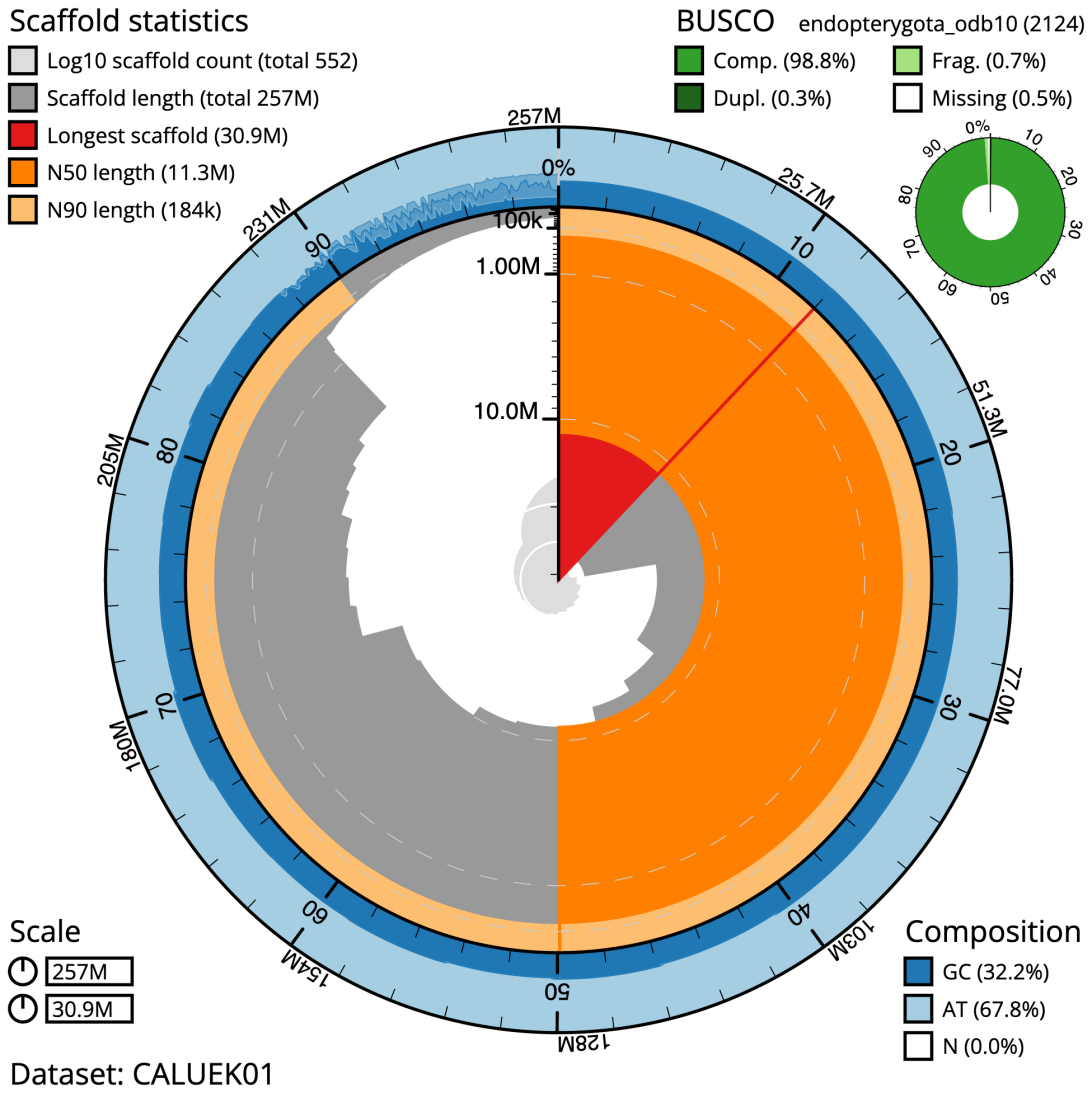
Genome assembly of *Nebria salina*, icNebSali1.1: metrics. The BlobToolKit Snailplot shows N50 metrics and BUSCO gene completeness. The main plot is divided into 1,000 size-ordered bins around the circumference with each bin representing 0.1% of the 256,740,106 bp assembly. The distribution of scaffold lengths is shown in dark grey with the plot radius scaled to the longest scaffold present in the assembly (30,902,303 bp, shown in red). Orange and pale-orange arcs show the N50 and N90 scaffold lengths (11,282,846 and 183,590 bp), respectively. The pale grey spiral shows the cumulative scaffold count on a log scale with white scale lines showing successive orders of magnitude. The blue and pale-blue area around the outside of the plot shows the distribution of GC, AT and N percentages in the same bins as the inner plot. A summary of complete, fragmented, duplicated and missing BUSCO genes in the endopterygota_odb10 set is shown in the top right. An interactive version of this figure is available at https://blobtoolkit.genomehubs.org/view/icNebSali1.1/dataset/CALUEK01/snail.

The final assembly has a total length of 256.7 Mb in 551 sequence scaffolds with a scaffold N50 of 11.3 Mb ( Table 1). Most (91.74%) of the assembly sequence was assigned to 21 chromosomal-level scaffolds, representing 20 autosomes, and the X sex chromosome. Chromosome-scale scaffolds confirmed by the Hi-C data are named in order of size ( Figure 2– Figure 5; Table 2). While not fully phased, the assembly deposited is of one haplotype. Contigs corresponding to the second haplotype have also been deposited. The mitochondrial genome was also assembled and can be found as a contig within the multifasta file of the genome submission.

**Table 1.** Genome data for *Nebria salina*, icNebSali1.1.

Project accession data		
Assembly identifier	icNebSali1.1	
Species	*Nebria salina*	
Specimen	icNebSali1	
NCBI taxonomy ID	878211	
BioProject	PRJEB45181	
BioSample ID	SAMEA7524273	
Isolate information	icNebSali1; female: thorax (genome sequencing); head (Hi-C scaffolding)	
Assembly metrics *		*Benchmark*
Consensus quality (QV)	49.3	*≥ 50*
*k*-mer completeness	99.99%	*≥ 95%*
BUSCO **	C:98.8%[S:98.4%,D:0.3%], F:0.7%,M:0.5%,n:2,124	*C ≥ 95%*
Percentage of assembly mapped to chromosomes	91.74%	*≥ 95%*
Sex chromosomes	X chromosome	*localised homologous pairs*
Organelles	Mitochondrial genome assembled	*complete single alleles*
Raw data accessions		
PacificBiosciences SEQUEL II	ERR6412374, ERR6590588	
10X Genomics Illumina	ERR6054889–ERR6054892	
Hi-C Illumina	ERR6054893	
Genome assembly		
Assembly accession	GCA_944039245.1	
*Accession of alternate haplotype*	GCA_944039265.1	
Span (Mb)	256.7	
Number of contigs	744	
Contig N50 length (Mb)	3.1	
Number of scaffolds	551	
Scaffold N50 length (Mb)	11.3	
Longest scaffold (Mb)	30.9	
**Genome annotation**		
Number of protein-coding genes	10,671	
Number of non-coding genes	3,153	
Number of transcripts	19,804	

* Assembly metric benchmarks are adapted from column VGP-2020 of “Table 1: Proposed standards and metrics for defining genome assembly quality” from ( Rhie *et al.*, 2021). ** BUSCO scores based on the endopterygota_odb10 BUSCO set using v5.3.2. C = complete [S = single copy, D = duplicated], F = fragmented, M = missing, n = number of orthologues in comparison. A full set of BUSCO scores is available at https://blobtoolkit.genomehubs.org/view/icNebSali1.1/dataset/CALUEK01/busco.

**Figure 3.**
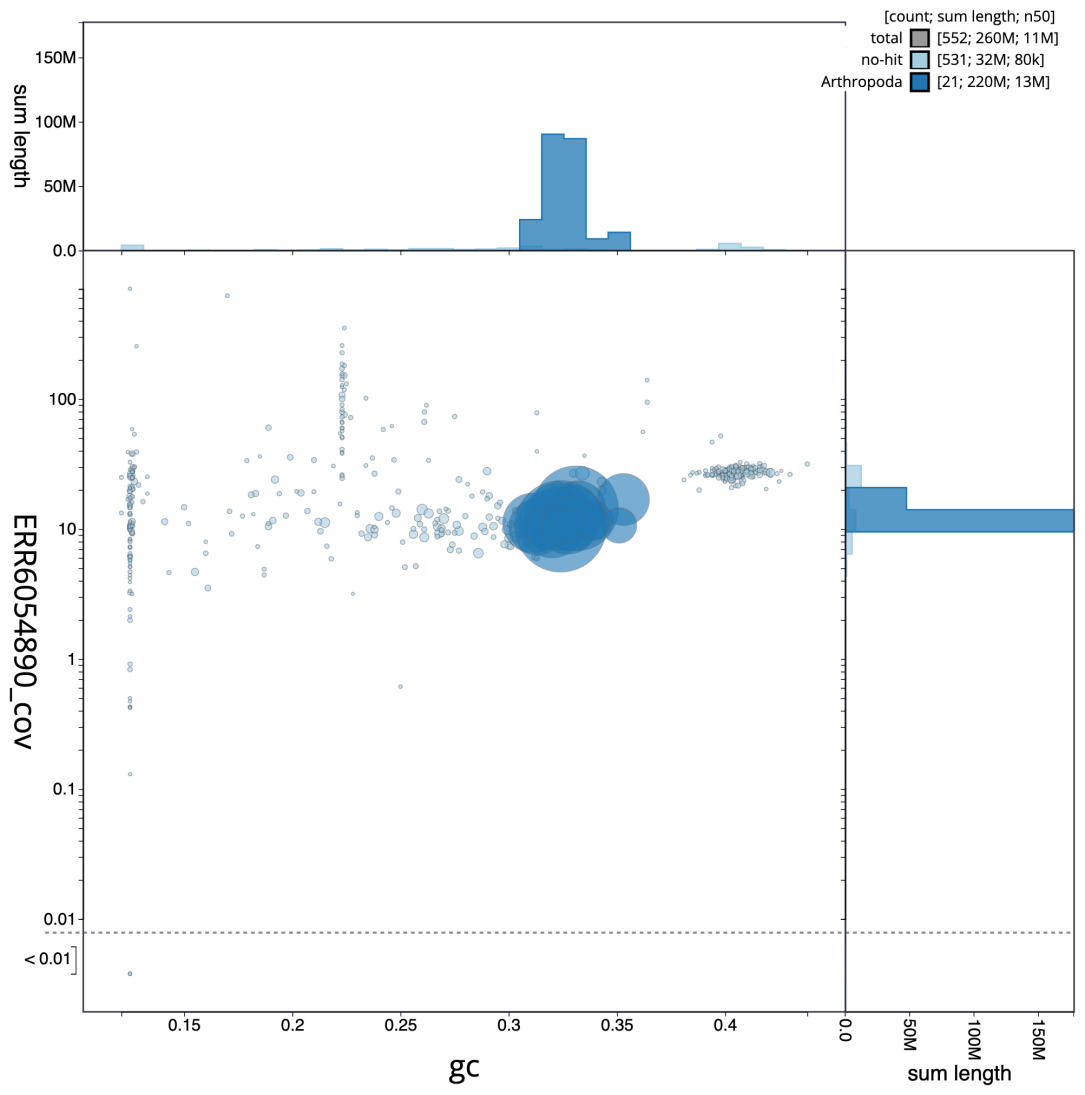
Genome assembly of *Nebria salina*, icNebSali1.1: GC coverage. BlobToolKit GC-coverage plot. Scaffolds are coloured by phylum. Circles are sized in proportion to scaffold length. Histograms show the distribution of scaffold length sum along each axis. An interactive version of this figure is available at https://blobtoolkit.genomehubs.org/view/icNebSali1.1/dataset/CALUEK01/blob.

**Figure 4.**
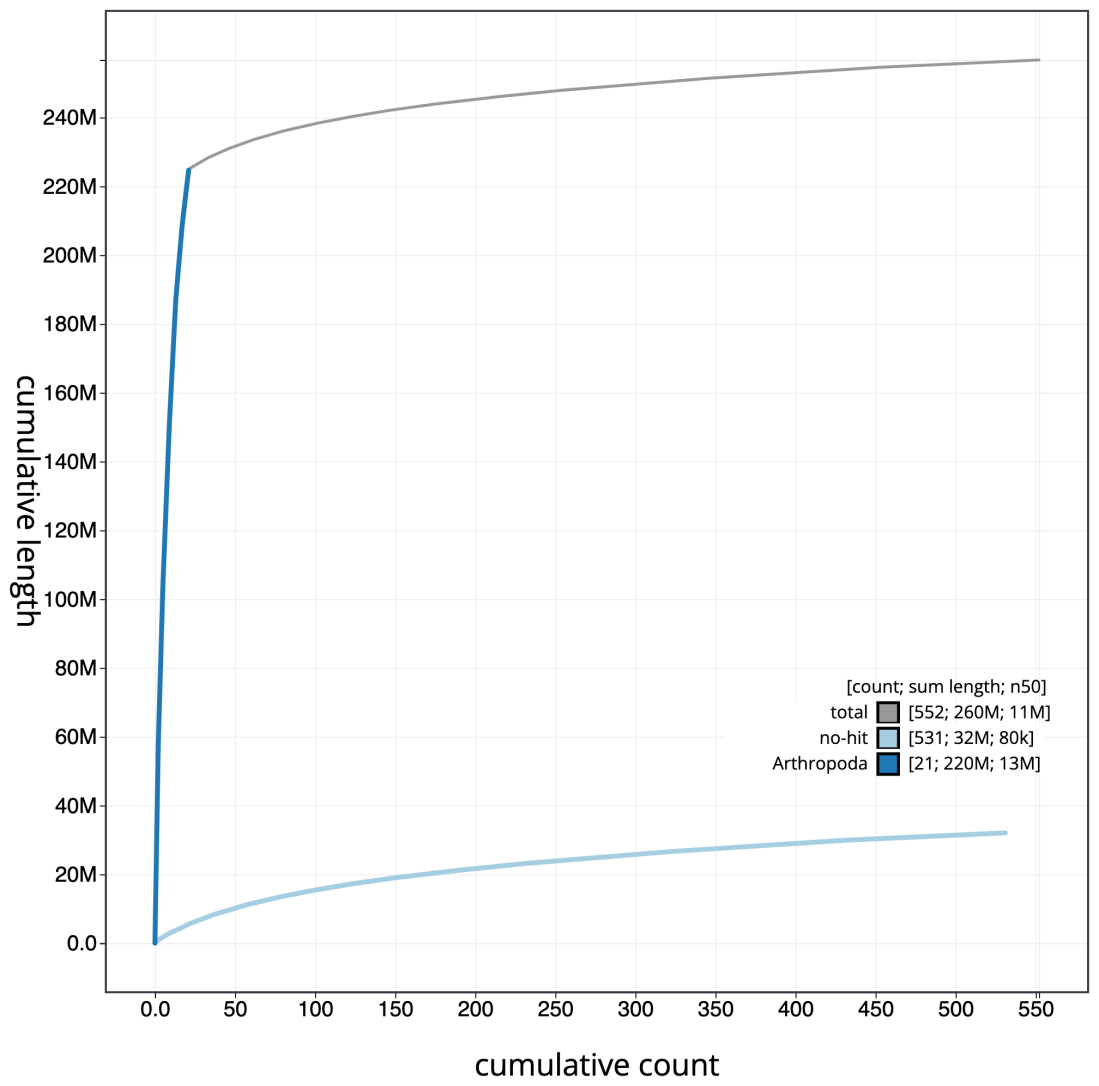
Genome assembly of *Nebria salina*, icNebSali1.1: cumulative sequence. BlobToolKit cumulative sequence plot. The grey line shows cumulative length for all scaffolds. Coloured lines show cumulative lengths of scaffolds assigned to each phylum using the buscogenes taxrule. An interactive version of this figure is available at https://blobtoolkit.genomehubs.org/view/icNebSali1.1/dataset/CALUEK01/cumulative.

**Figure 5.**
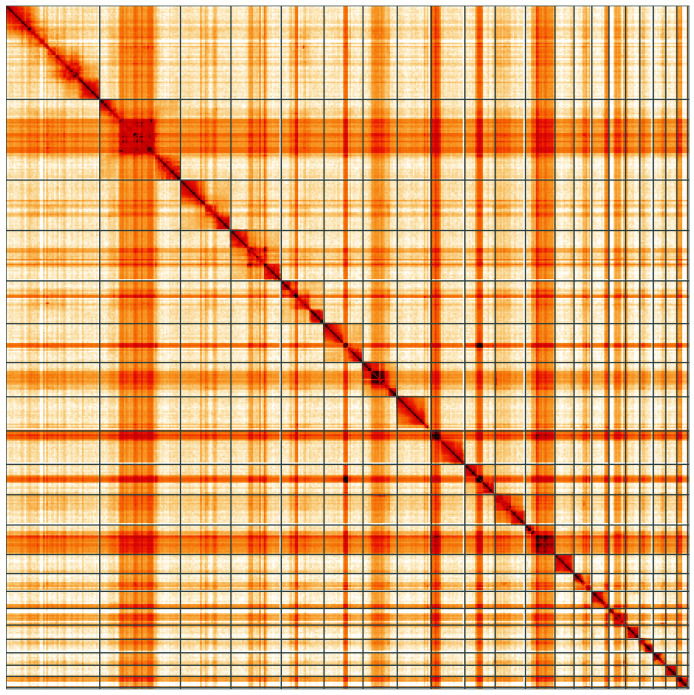
Genome assembly of *Nebria salina*, icNebSali1.1: Hi-C contact map. Hi-C contact map of the icNebSali1.1 assembly, visualised using HiGlass. Chromosomes are shown in order of size from left to right and top to bottom. An interactive version of this figure may be viewed at https://genome-note-higlass.tol.sanger.ac.uk/l/?d=eQJxQM3JQcKLF5KpyGKRZA.

**Table 2.** Chromosomal pseudomolecules in the genome assembly of *Nebria salina*, icNebSali1.

INSDC accession	Chromosome	Size (Mb)	GC%
OX063268.1	1	26.58	33.1
OX063269.1	2	11.02	33.2
OX063270.1	3	16.63	32
OX063271.1	4	16.61	32.3
OX063272.1	5	14.09	32.7
OX063273.1	6	12.84	31.1
OX063274.1	7	11.28	33
OX063275.1	8	11.25	31.3
OX063276.1	9	9.99	32.2
OX063277.1	10	9.98	32.9
OX063278.1	11	9.74	35.3
OX063279.1	12	6.28	31.7
OX063280.1	13	5.84	32.4
OX063281.1	14	5.65	32.7
OX063282.1	15	5.48	33.9
OX063283.1	16	4.69	32.6
OX063284.1	17	4.41	35.1
OX063285.1	18	4.12	32.3
OX063286.1	19	3.64	33.9
OX063287.1	20	3.65	32.8
OX063288.1	X	30.9	32.4
OX063289.1	MT	0.02	17
-	unplaced	32.03	29.4

The estimated Quality Value (QV) of the final assembly is 49.3 with *k*-mer completeness of 99.95%, and the assembly has a BUSCO v5.3.2 completeness of 98.8% (single = 98.4%, duplicated = 0.3%), using the endopterygota_odb10 reference set ( *n* = 2,124).

Metadata for specimens, spectral estimates, sequencing runs, contaminants and pre-curation assembly statistics can be found at https://links.tol.sanger.ac.uk/species/878211.

## Genome annotation report

The GCA_944039245.1 genome was annotated using the Ensembl rapid annotation pipeline ( Table 1; https://rapid.ensembl.org/Nebria_salina_GCA_944039245.1/Info/Index). The resulting annotation includes 19,804 transcribed mRNAs from 10,671 protein-coding and 3,153 non-coding genes.

## Methods

### Sample acquisition and nucleic acid extraction

One adult *Nebria salina* (icNebSali1) was hand-picked from Wigmore Park, Luton, England (51.88, –0.37) on 20 May 2020 by Olga Sivell. It was identified by Duncan Sivell following Luff (2007) and Walters (2010). The specimen was snap-frozen using dry ice. The tissue samples taken from it were stored in a CoolRack prior to genome sequencing.

DNA was extracted at the Tree of Life laboratory, Wellcome Sanger Institute (WSI). The icNebSali1 sample was weighed and dissected on dry ice with head tissue set aside for Hi-C sequencing. Thorax tissue was disrupted using a Nippi Powermasher fitted with a BioMasher pestle. High molecular weight (HMW) DNA was extracted using the Qiagen MagAttract HMW DNA extraction kit. Low molecular weight DNA was removed from a 20 ng aliquot of extracted DNA using the 0.8X AMpure XP purification kit prior to 10X Chromium sequencing; a minimum of 50 ng DNA was submitted for 10X sequencing. HMW DNA was sheared into an average fragment size of 12–20 kb in a Megaruptor 3 system with speed setting 30. Sheared DNA was purified by solid-phase reversible immobilisation using AMPure PB beads with a 1.8X ratio of beads to sample to remove the shorter fragments and concentrate the DNA sample. The concentration of the sheared and purified DNA was assessed using a Nanodrop spectrophotometer and Qubit Fluorometer and Qubit dsDNA High Sensitivity Assay kit. Fragment size distribution was evaluated by running the sample on the FemtoPulse system.

### Sequencing

Pacific Biosciences HiFi circular consensus and 10X Genomics read cloud DNA sequencing libraries were constructed according to the manufacturers’ instructions. DNA sequencing was performed by the Scientific Operations core at the WSI on Pacific Biosciences SEQUEL II (HiFi) and HiSeq X Ten (10X) instruments. Hi-C data were also generated from head tissue of icNebSali1 using the Arima v2 kit and sequenced on the Illumina NovaSeq 6000 instrument.

### Genome assembly, curation and evaluation

Assembly was carried out with Hifiasm ( Cheng *et al.*, 2021) and haplotypic duplication was identified and removed with purge_dups ( Guan *et al.*, 2020). One round of polishing was performed by aligning 10X Genomics read data to the assembly with Long Ranger ALIGN, calling variants with FreeBayes ( Garrison & Marth, 2012). The assembly was then scaffolded with Hi-C data ( Rao *et al.*, 2014) using YaHS ( Zhou *et al.*, 2023) SALSA2 ( Ghurye *et al.*, 2019). The assembly was checked for contamination and corrected using the gEVAL system ( Chow *et al.*, 2016) as described previously ( Howe *et al.*, 2021). Manual curation was performed using gEVAL, HiGlass ( Kerpedjiev *et al.*, 2018) and Pretext ( Harry, 2022). The mitochondrial genome was assembled using MitoHiFi ( Uliano-Silva *et al.*, 2022), which runs MitoFinder ( Allio *et al.*, 2020) or MITOS ( Bernt *et al.*, 2013) and uses these annotations to select the final mitochondrial contig and to ensure the general quality of the sequence. To evaluate the assembly, MerquryFK was used to estimate consensus quality (QV) scores and *k*-mer completeness ( Rhie *et al.*, 2020). The genome was analysed within the BlobToolKit environment ( Challis *et al.*, 2020) and BUSCO scores ( Manni *et al.*, 2021; Simão *et al.*, 2015) were calculated. Table 3 contains a list of software tool versions and sources.

**Table 3.** Software tools: versions and sources.

Software tool	Version	Source
BlobToolKit	4.0.7	https://github.com/blobtoolkit/blobtoolkit
BUSCO	5.3.2	https://gitlab.com/ezlab/busco
FreeBayes	1.3.1-17-gaa2ace8	https://github.com/freebayes/freebayes
gEVAL	N/A	https://geval.org.uk/
Hifiasm	0.12	https://github.com/chhylp123/hifiasm
HiGlass	1.11.6	https://github.com/higlass/higlass
Long Ranger ALIGN	2.2.2	https://support.10xgenomics.com/genome-exome/ software/pipelines/latest/advanced/other-pipelines
Merqury	MerquryFK	https://github.com/thegenemyers/MERQURY.FK
MitoHiFi	2	https://github.com/marcelauliano/MitoHiFi
PretextView	0.2	https://github.com/wtsi-hpag/PretextView
purge_dups	1.2.3	https://github.com/dfguan/purge_dups
SALSA	2.2	https://github.com/salsa-rs/salsa

### Genome annotation

The Ensembl gene annotation system ( Aken *et al.*, 2016) was used to generate annotation for the *Nebria salina* assembly (GCA_944039245.1). Annotation was created primarily through alignment of transcriptomic data to the genome, with gap filling via protein-to-genome alignments of a select set of proteins from UniProt ( UniProt Consortium, 2019).

### Ethics and compliance issues

The materials that have contributed to this genome note have been supplied by a Darwin Tree of Life Partner. The submission of materials by a Darwin Tree of Life Partner is subject to the Darwin Tree of Life Project Sampling Code of Practice. By agreeing with and signing up to the Sampling Code of Practice, the Darwin Tree of Life Partner agrees they will meet the legal and ethical requirements and standards set out within this document in respect of all samples acquired for, and supplied to, the Darwin Tree of Life Project. All efforts are undertaken to minimise the suffering of animals used for sequencing. Each transfer of samples is further undertaken according to a Research Collaboration Agreement or Material Transfer Agreement entered into by the Darwin Tree of Life Partner, Genome Research Limited (operating as the Wellcome Sanger Institute), and in some circumstances other Darwin Tree of Life collaborators.

## Data Availability

European Nucleotide Archive: *Nebria salina*. Accession number PRJEB45181; https://identifiers.org/ena.embl/PRJEB45181. ( Wellcome Sanger Institute, 2022) The genome sequence is released openly for reuse. The *Nebria salina* genome sequencing initiative is part of the Darwin Tree of Life (DToL) project. All raw sequence data and the assembly have been deposited in INSDC databases. Raw data and assembly accession identifiers are reported in Table 1.

## Author information

Members of the Natural History Museum Genome Acquisition Lab are listed here: https://doi.org/10.5281/zenodo.4790043.

Members of the Darwin Tree of Life Barcoding collective are listed here: https://doi.org/10.5281/zenodo.4893703.

Members of the Wellcome Sanger Institute Tree of Life programme are listed here: https://doi.org/10.5281/zenodo.4783585.

Members of Wellcome Sanger Institute Scientific Operations: DNA Pipelines collective are listed here: https://doi.org/10.5281/zenodo.4790455.

Members of the Tree of Life Core Informatics collective are listed here: https://doi.org/10.5281/zenodo.5013541.

Members of the Darwin Tree of Life Consortium are listed here: https://doi.org/10.5281/zenodo.4783558.
