# Supplementary material for: The genome sequence of a carabid beetle, Nebria salina (Fairmaire & Laboulbène, 1854)
Source: Wellcome Open Res. 2023 Jun 14;8:247. [Version 1] doi: 10.12688/wellcomeopenres.19372.1 (PMC10774627; doi:10.12688/wellcomeopenres.19372.1)
Supplement: Peer review history

# Reviewer report: Reviewer response for version 1

**DOI:** 10.21956/wellcomeopenres.21459.r71438

**Published:** 2024-01-08

**Authors:** XiaoLong Liu

**Affiliations:** 1 Hubei University, Wuhan, China

In this work, the methods part includes DNA extraction, sequencing, genome assembly, genome annotation, and so on, and the work is appropriate, and the details are sufficient. The genome sequence is released openly and clearly presented. The raw sequence and assembly data have been deposited in INSDC databases and listed in the manuscript.

 During the review process, I identified one issue,

 The genome sequence of *Nebria salina*, used one female. Readers who are not proficient in this field, may have a question, why not use the male and the differences in the genomes of male and female. In the “Background” part, it is better to add the pattern of sexual differentiation and mention the X chromosome.

Are sufficient details of methods and materials provided to allow replication by others?

Yes

Is the rationale for creating the dataset(s) clearly described?

Yes

Are the datasets clearly presented in a useable and accessible format?

Yes

Are the protocols appropriate and is the work technically sound?

Yes

Reviewer Expertise:

Insect biochemistry and physiology, Transcriptomics

I confirm that I have read this submission and believe that I have an appropriate level of expertise to confirm that it is of an acceptable scientific standard.

# Reviewer report: Reviewer response for version 1

**DOI:** 10.21956/wellcomeopenres.21459.r60416

**Published:** 2023-07-10

**Authors:** Ladislav Bocak

**Affiliations:** 1 Biodiversity & Molecular Evolution, Czech Advanced Technology and Research Institute, Olomouc, Czech Republic

Sivell & Sivell have presented a chromosome-level genome assembly of an individual female *Nebria salina*, a carabid beetle from the Carabidae family in Adephaga. The genome sequence data has been scaffolded into 21 chromosomal pseudomolecules, including the assembled X sex chromosome. While some assembly metrics values fall slightly below the benchmarks, the study significantly contributes to the exploration of the earliest evolution of beetles, which represent the largest radiation of animals on Earth.

 To enhance the overall understanding of *Nebria's* taxonomic position, it is recommended that the authors provide a more detailed discussion, even if it means sacrificing some aspects of bionomics and species-level comparisons. Additionally, it would be beneficial to include information on other available genomes of the Adephaga suborder to provide a broader context for the findings.

 The research presentation is clear and important for future advancements in the field.

Are sufficient details of methods and materials provided to allow replication by others?

Yes

Is the rationale for creating the dataset(s) clearly described?

Yes

Are the datasets clearly presented in a useable and accessible format?

Yes

Are the protocols appropriate and is the work technically sound?

Yes

Reviewer Expertise:

Entomology, phylogeny

I confirm that I have read this submission and believe that I have an appropriate level of expertise to confirm that it is of an acceptable scientific standard.
